# Analytical power calculations for structural equation modeling: A tutorial and Shiny app

**DOI:** 10.3758/s13428-020-01479-0

**Published:** 2020-11-02

**Authors:** Suzanne Jak, Terrence D. Jorgensen, Mathilde G. E. Verdam, Frans J. Oort, Louise Elffers

**Affiliations:** 1 Methods and Statistics, Research Institute of Child Development and Education, University of Amsterdam, Nieuwe Achtergracht 127, 1018 WS Amsterdam, The Netherlands; 2 Methodology and Statistics, Institute of Psychology, Leiden University, Leiden, The Netherlands; 3 Educational Sciences, Child Development and Education, University of Amsterdam, Amsterdam, The Netherlands

**Keywords:** Power analysis, Structural equation modeling, Root mean square error of approximation, Likelihood ratio test, Sample size planning

## Abstract

Conducting a power analysis can be challenging for researchers who plan to analyze their data using structural equation models (SEMs), particularly when Monte Carlo methods are used to obtain power. In this tutorial, we explain how power calculations without Monte Carlo methods for the χ^2^ test and the RMSEA tests of (not-)close fit can be conducted using the Shiny app “power4SEM”. power4SEM facilitates power calculations for SEM using two methods that are not computationally intensive and that focus on model fit instead of the statistical significance of (functions of) parameters. These are the method proposed by Satorra and Saris (Psychometrika 50(1), 83–90, 1985) for power calculations of the likelihood ratio test, and that described by MacCallum, Browne, and Sugawara (Psychol Methods 1(2) 130–149, 1996) for RMSEA-based power calculations. We illustrate the use of power4SEM with examples of power analyses for path models, factor models, and a latent growth model.

Before any quantitative study is conducted, one should evaluate how large the sample should be for the study to be adequately powered (Cohen, 1992). That is, there should be a fair chance to reject the null hypothesis (H_0_) if it is indeed false. When statistical power is too low to detect a meaningful effect, a study would essentially waste data on type II errors. When the power is approximately 100%, a researcher may be wasting often expensive resources because the effect of interest could have been detected with a smaller sample size. To prevent under- or overpowered studies, researchers need to calculate the minimum sample size required to sufficiently minimize the chance of type II errors before they start collecting data. For simple analyses such as *t* tests or simple regression models, there are user-friendly tools to calculate statistical power, such as G*Power (Erdfelder, Faul & Buchner, 1996) or the R (R Core Team, 2019) package pwr (Champely, 2018). However, for researchers who intend to apply structural equation modeling to test their hypotheses, conducting a power analysis is more challenging.

There are three ways to calculate power for structural equation models (SEMs). One is by performing a Monte Carlo simulation study (Muthén & Muthén, 2002). This is a computationally intensive method in which a researcher generates a large number of data sets from a population model corresponding to an alternative hypothesis (H_1_), fits the model corresponding to the null hypothesis (H_0_) to all generated data sets, and calculates the proportion of data sets for which the statistic or parameter of interest (e.g. χ^2^ value, regression coefficient, or indirect effect) is statistically significant. This method provides an empirical estimate of power. For instructions on how to conduct such a study, see the articles by Muthén and Muthén (2002), Schoemann, Boulton, and Short (2017), or Wang and Rhemtulla (2020). In this tutorial we focus on two methods that are not computationally intensive and that focus on model fit instead of the statistical significance of (functions of) parameters: the method introduced by Satorra and Saris (1985) for power calculations of the likelihood ratio test (LRT), and that by MacCallum, Browne, and Sugawara (1996) for the calculation of root mean square error of approximation (RMSEA)-based power. Because the original articles in which the methods are described are relatively technical, applying the methods may not be straightforward for researchers outside the field of statistics. In this paper we aim to provide a more accessible explanation of power calculations for SEM, using the two abovementioned methods, for researchers who need to conduct power analyses but who are less familiar with the technical side of such analyses. We provide power4SEM, which is an interactive Shiny app, available through (https://sjak.shinyapps.io/power4SEM/)^1^, that can be used to calculate both the power for a given sample size, model, and significance level, and the necessary sample size to obtain a desired power level given the model and significance level.

Our aim is to provide software and a tutorial directly targeted to computationally non-intensive power calculations for SEM. We are not the first to try to make SEM-based power calculations more accessible. Miles (2003) is a useful resource for an introduction to the theory behind the Satorra and Saris (1985) method. Zhang and Yuan (2018) developed WebPower, which is a general software tool for statistical power analysis, including power analyses for SEM. They provide a manual for the software and a technical report for the methods used. Moshagen and Erdfelder’s work (2016) led to the development of an R package and Shiny app called semPower (Moshagen 2018), which focuses on “compromise power.” Compromise power involves balancing the risk of committing type I and type II errors. However, the app can also be used to do the power analyses as described in this tutorial.

In comparison with the work by Zhang and Yuan (2018) and Moshagen and Erdfelder (2016), our tutorial is targeted at an audience with slightly less statistical knowledge. What our work adds to Miles (2003) is the discussion of RMSEA-based power analysis, and the addition of the software with instructions and examples of how to apply it. This tutorial and software therefore supplement the existing literature on SEM-based power analysis. With multiple recourses available, researchers can benefit from the perspectives of different authors explaining the same technique, or choose the one that best fits their needs.

This tutorial is aimed at users with a basic knowledge of SEM, who are able to fit models in the R (R Core Team, 2019) package lavaan (Rosseel, 2012)^2^. In the next section, we briefly introduce the concept of statistical power. We then provide a nontechnical explanation of the method by Satorra and Saris (1985), which we will call χ^2^-based power, followed by example analyses in power4SEM. Next, we explain the method by MacCallum et al. (1996), which we will call RMSEA-based power, again followed by example analyses in power4SEM.

## Statistical power

A statistical test can be applied to obtain the probability (the *p* value) of finding a test statistic at least as extreme as the one from the given sample, given that the H_0_ about the population value is true. When the *p* value is smaller than the chosen significance level (e.g., α = .05), then H_0_ will be rejected in favor of the alternative hypothesis (H_1_). When the H_0_ is not rejected while H_0_ is actually false (so H_1_ is true), one is making a type II error, and the probability of doing so is denoted by β. It is therefore important to know the probability of rejecting a false H_0_, which is the power (1 − β) of a statistical test. Table 1 presents an overview of the relations between truth/falseness of the null hypothesis and outcomes of the test.

**Table 1:** Overview of the relations between truth/falseness of the null hypothesis and outcomes of the test

			**True hypothesis**	
			**H**_**0**_	**H**_**1**_
**Outcome** **of** **statistical** **test**		**H**_**0**_ **rejected**	Type I error (α)	Power (1 − β)
		**H**_**0**_ **not rejected**	Correct inference (1 − α)	Type II error (β)

In applied hypothesis testing, H_1_ represents a range of values. For example, H_1_ may be that two means are unequal, or that a regression coefficient is larger than zero. However, to evaluate the power of a statistical test, researchers have to determine a *specific value* for H_1_. In the simple example of a *t* test, one may calculate the power to reject the H_0_ of zero difference between two group means, given that in the population there is a mean difference of 0.5 standard deviations between groups (i.e., the standardized effect size; Cohen’s *d* = 0.50, representing a “medium-sized” effect^3^). For a given sample size (*N*) and significance level, the larger the difference between the null-hypothesized effect size and the effect size under H_1_, the larger the statistical power. So, for example, the statistical power to detect an effect size of *d* = 0.80 (representing a “large” effect) will be larger than the statistical power to detect an effect size of *d* = 0.50. The statistical power also increases with increasing sample size and with increasing significance level (but the latter also increases the probability of making a type I error). Note that in this example, the hypotheses refer to only one parameter: the difference between two group means. In SEM, many parameters are involved (e.g. direct effects, factor loadings, residual variances), making power calculations more complex.

## χ^2^-based power

Satorra and Saris (1985) developed a method for estimating the power of the LRT (i.e., a SEM’s χ^2^ fit statistic) in SEM. This method can be used to estimate the power to detect overall misspecification of SEMs, and to estimate the power to detect misspecification due to specific parameters. We will first discuss the power related to overall fit of the model, and then explain how the same procedure can be used for power calculations related to specific parameters.

### Theoretical background: Power to reject overall exact model fit

At the population level, the variables in a SEM may be related to each other. The population covariance matrix between the variables is denoted by **Σ**_population_. A researcher who plans to use SEM specifies a model that presumably explains the variances and covariances between the variables. The parameters in that model (for example, factor loadings, factor (co)variances, and residual variances in a factor model) lead to a so-called model-implied covariance matrix, denoted by **Σ**_model_. If the researcher specified the correct model, then the specified model indeed gives rise to the population covariance matrix, and **Σ**_population_ = **Σ**_model_. If the specified model is not exactly correct, there is another model leading to **Σ**_population_, resulting in a discrepancy between **Σ**_model_ and **Σ**_population_, so that **Σ**_population_ ≠ **Σ**_model_. The discrepancy between **Σ**_population_ and **Σ**_model_ is denoted by F_0_.

The χ^2^ test of overall fit in SEM tests whether the hypothesized model fits exactly in the population—that is, the H_0_ that the population discrepancy F_0_ is zero. When H_0_ is true, the expected value of the χ^2^ statistic equals the expected sampling error, which is equal to the degrees of freedom (*df*) of a model. The *df* of a model can be calculated by counting the number of observed statistics *p* (the number of unique elements in the observed covariance matrix and mean vector of the variables) and the number of model parameters to be estimated, *q*. The model’s *df* is then equal to *df = p* − *q.* Calculation of a model’s degrees of freedom will be illustrated in the example analysis in the next section.

Fitting the hypothesized model to data leads to an observed χ^2^ statistic. The *p* value associated with the observed χ^2^ statistic and the model’s *df* gives the probability of observing a sample discrepancy at least as large as the observed one, when any discrepancy is solely due to random sampling error. When this probability is smaller than the nominal α level, H_0_ is rejected, implying that the model does not hold exactly in the population. In other words, we conclude that the model is misspecified.

The H_0_ thus represents the case that the model fits the data exactly. When this is true, the expected χ^2^ value will be equal to the expected sampling error, i.e. with E() denoting the expected value: E(χ^2^) = E(sampling error) = *df*. The H_1_ is that the model does not fit the data exactly. When H_1_ is true but the (misspecified) H_0_ model is fit to the data, the test statistic also asymptotically follows a χ^2^ distribution (assuming multivariate normality and limited misfit), but with a larger mean and larger sampling variance. As a result, the distribution of the χ^2^ statistic under H_1_ lies more to the right, and is more spread out, than the distribution of the χ^2^ statistic under H_0_. The expected χ^2^ value under H_1_ consists not only of discrepancies due to sampling error, but also discrepancies due to misspecification, i.e., E(χ^2^) = E(sampling error) + E(misspecification error). The expected misspecification error is called the noncentrality parameter, denoted by λ. Therefore, under H_1_, the expected χ^2^ statistic equals *df* + λ. The exact size of λ depends on the population discrepancy F_0_ and the sample size (see Moshagen & Erdfelder, 2016):

1 $$\uplambda =n\times {\mathrm{F}}_0,$$

where *n* = *N* under normal-theory^4^ maximum likelihood estimation.

To summarize, under H_0_ the test statistic follows a central χ^2^ distribution, with an expected value (i.e., mean) equal to its *df* parameter, and sampling variance equal to 2 × *df*. Under H_1_, the test statistic follows a χ^2^ distribution that is *noncentral*, with a mean equal to its *df* plus its noncentrality parameter λ—a nonnegative number that quantifies the degree of misspecification error—and sampling variance equal to 2*df* + 4λ (i.e., greater misspecification leads to more variability between replications of a study). Table 2 provides an overview of the hypotheses, models, and distributions associated with H_0_ and H_1_.

**Table 2:** Overview of the hypotheses, models, and distributions associated with H_0_ and H_1_ of the overall χ^2^ test

	H_0_	H_1_
Hypothesis	**Σ**_population_ = **Σ**_model_	**Σ**_population_ ≠ **Σ**_model_
Model leading to **Σ**_population_	Model H_0_	Model H_1_
Value of population discrepancy F_0_	F_0_ = 0	F_0_ > 0
Distribution of test statistic	Central χ^2^	Noncentral χ^2^
Mean of test statistic	*df*	*df* + λ

**Fig. 1:**
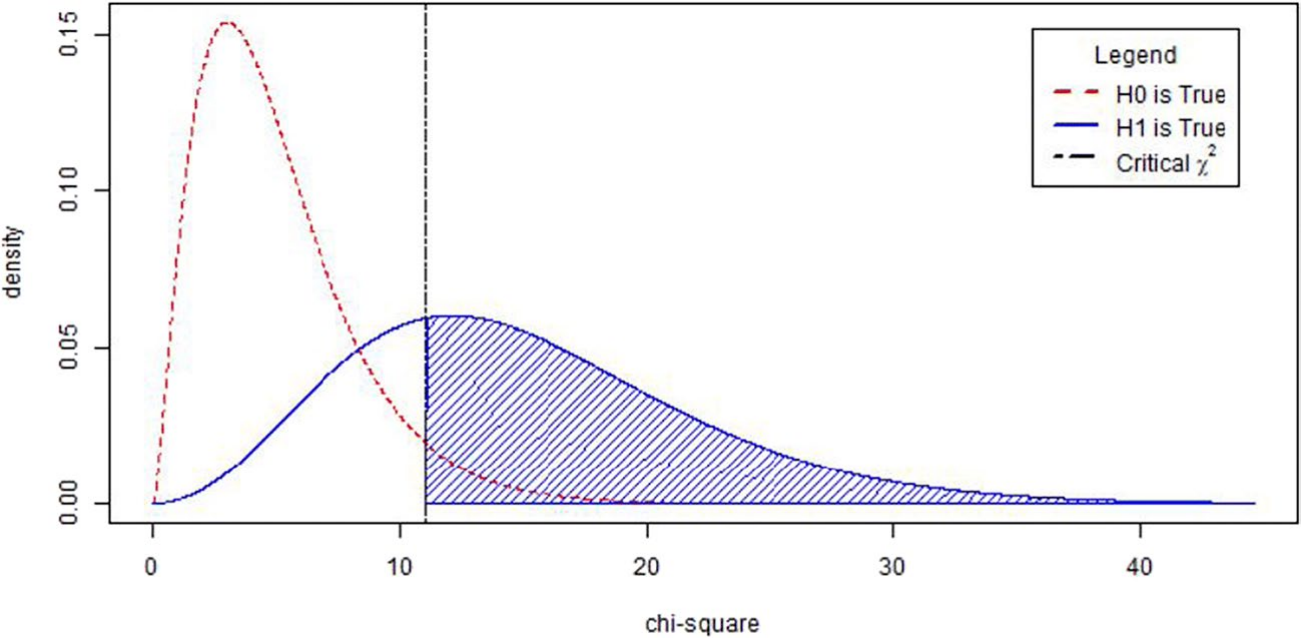
A central χ^2^ distribution with *df* = 5 (dashed red line), and a noncentral χ^2^ distribution with *df* = 5 and λ = 10 (blue solid line). The shaded area corresponds to the statistical power with α = 0.05

Figure 1 shows a central χ^2^ distribution with *df* = 5 in red, and a noncentral χ^2^ distribution with *df* = 5 and λ = 10 in blue. The noncentral χ^2^ distribution is the χ^2^ distribution associated with H_1_. The vertical line indicates the critical χ^2^ value under the central χ^2^ distribution that is associated with the H_0_ with α = .05. The H_0_ will only be rejected if the observed χ^2^ value is larger than the critical value. The blue area under the H_1_ curve then shows the statistical power: the probability of rejecting H_0_ given that H_1_ is true. This probability is easy to obtain if one knows the two distributions of the test statistic under H_0_ and H_1_. The most challenging part of computing χ^2^-based power in SEM is obtaining the noncentrality parameter associated with a specific H_1_.

Satorra and Saris (1985) showed that in order to obtain the noncentrality parameter for the χ^2^ test in SEM, one can fit the H_0_ model to covariances (and means) implied by the population model under H_1_. Because the model is fit to population moments, the sampling error is eliminated from the model (E(sampling error) = 0). All resulting discrepancies therefore arise from misspecification error, so that

2 $$\mathrm{E}\left({\upchi}^2\right)=0+\mathrm{E}\left(\mathrm{misspecification}\ \mathrm{error}\right)=0+\uplambda .$$

The χ^2^ value obtained in this way is therefore the noncentrality parameter λ under H_1_.

Practically, a researcher performing a SEM power analysis first has to formulate the H_0_ model. This is the model that the researcher thinks is the correct model. Next, the researcher has to think about a situation in which the H_0_ model should be rejected. That is, they have to define what H_1_ actually represents, by formulating a model with one or more additional parameters that are not zero. They then calculate the statistical power to reject the H_0_ model when H_1_ is true. Although conceptually it is easier to think about the H_0_ model first, and then define how the H_0_ model might be wrong (or what misspecification one wants to be able to detect with sufficient power), in order to perform power calculations, one has to specify the H_1_ model first, followed by the H_0_ model.

The following steps are used to obtain the statistical power (Saris & Satorra, 1993):

- **Step 1**: Calculate the model-implied population covariance matrix under the alternative-hypothesized model (Model H_1_). The calculated covariance matrix is treated as population data in Step 2.
- **Step 2**: Fit the null-hypothesized model (Model H_0_) to the model-implied covariance matrix from Step 1.
- **Step 3**: Use the χ^2^ value from Step 2 as the noncentrality parameter λ to calculate the statistical power.

We will illustrate these three steps with power analyses for the overall fit of a path model.

### Example 1: Calculating the power of the χ^2^ test for overall fit of a path model

**Fig. 2:**
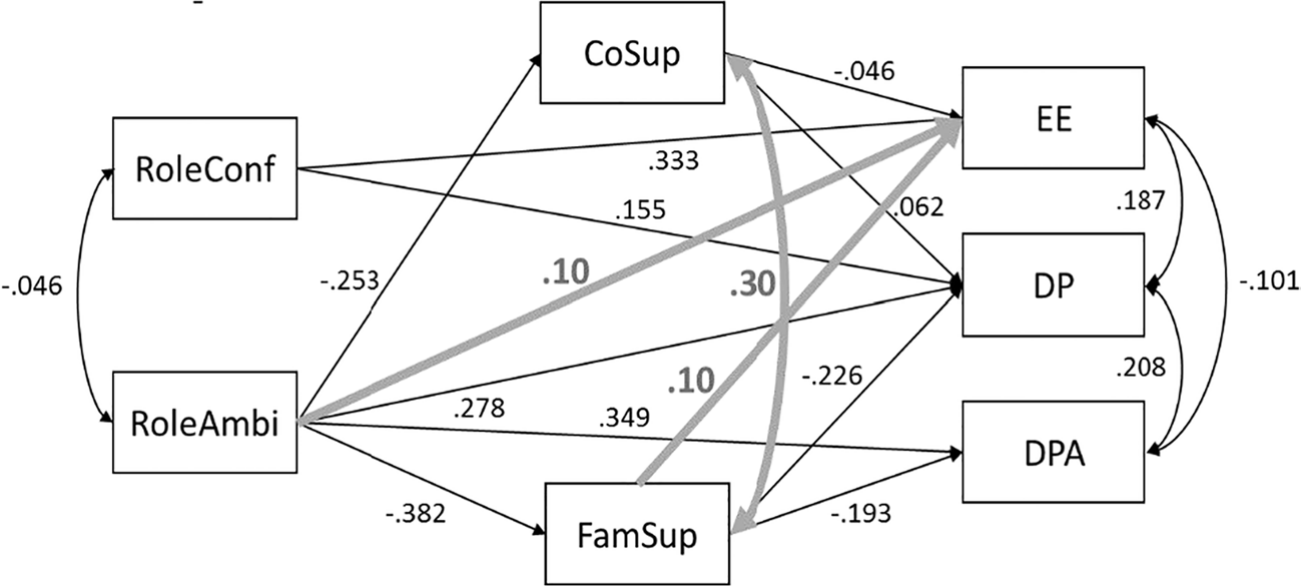
Path model for the example power calculations, with population values for H_0_ based on empirical results and three extra parameters for H_1_. The variables and population values stem from Ma et al. (2020). RoleConf = role conflict, RoleAmbi = role ambiguity, CoSup = coworker support, FamSup = family support, EE = emotional exhaustion, DP = depersonalization, DPA = decreased personal accomplishment. Population values for (residual) variances are not depicted: RoleConf: 1, RoleAmbi: 1 CoSup: .936, FamSup: .853, EE: .887, DP: .812, DPA: .789

As an example, we use the path model that was analyzed by Ma et al. (2020). It evaluates the effects of role conflict, role ambiguity, coworker support, and family support on three outcomes: emotional exhaustion (EE), depersonalization (DP), and decreased personal accomplishment (DPA). This path model is shown in Fig. 2, using the thinner black lines (so the thicker gray lines should be ignored for now). The model contains seven variances, four covariances, and 10 regression coefficients to be estimated, leading to a total of 21 parameters. The number of unique elements in the observed covariance matrix equals (7 × 8)/2 = 28. Thus, *df* = 28 − 21 = 7. With a significance level of α = .05, exact fit of this model would be rejected if the χ^2^ value obtained were larger than the critical value of a χ^2^ distribution with *df* = 7 and α = .05, which equals χ^2^ = 14.067. In order to calculate the power of the overall χ^2^ test, we follow the three steps as outlined above.

- **Step 1 -** We have to specify an H_1_ model that contains more parameters than the model to be tested (H_0_). We have to specify the population values for all parameters in the model, including the parameters that are also included in the model under H_0_. For this example we use the standardized parameter estimates obtained by Ma et al. (2019) as population values for the parameters that are also included in the H_0_ model. Figure 2 shows the path model with the smaller black lines representing these population parameters. In general, it may be convenient to specify the parameter values in standardized form, so one can base values on the guidelines regarding small, medium, and large effects in the appropriate research domain. Next, we have to specify the parameters that are present under H_1_, but not under H_0_. These parameters define exactly how the model under H_0_ is misspecified. As there are many options for defining H_1_, it may require quite some deliberation to decide what the exact misspecification should entail. In principle, we would advise researchers to think about the parameters that should really lead to rejection of H_0_ if they are not zero. Regarding the value of these parameters, our recommendation would be to choose the minimum value that would be of interest. In our example, we added two small effects to the model associated with H_1_: an effect of .10 for role ambiguity on EE, and an effect of .10 for family support on EE. In addition, we added a covariance between the residuals of family support and coworker support of .30. Note that specifying only these three extra parameters implies that we chose population values of zero for the rest of the parameters, such as the effect of role conflict on DPA. Figure 2 shows the population values of all parameters under H_1_, with the extra parameters indicated in thicker gray lines. The goal of step 1 of the procedure is to generate population data based on H_1_. If one wants to generate data in R, one can for example specify the population values in designated matrices and use matrix algebra to do so. Appendix 1 provides the R code to calculate the model-implied covariance matrix with matrix algebra for this example. However, the power4SEM app lets users specify the model in lavaan syntax with all fixed parameters, and will do these calculations behind the scenes using functions from the semTools package (Jorgensen, Pornprasertmanit, Schoemann & Rosseel, 2020). Below, we show the lavaan syntax that specifies our example model under H_1_.

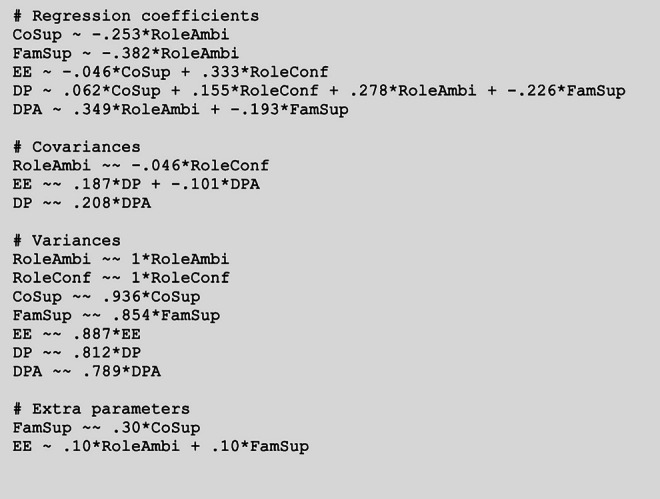

All parameters are fixed at the (chosen) population values using the multiplication operator. For example, the population direct effect of RoleAmbi on CoSup is specified as being −.253 using “CoSup ~ -.253*RoleAmbi.” In the app, a graphical display of the model will appear at the right side of the dialog box. This figure is created using the semPlot package (Epskamp, 2019). Although the outline of these figures may not always be optimal, especially with larger models, this graphical display can be used to check whether all population values are indeed specified as fixed parameters. If the model syntax still contains unspecified/free direct effects or (co)variances, these will be displayed in red.

Note that we started by using the standardized parameter values as reported by Ma et al., to ensure meaningful interpretation of the size of parameters However, by adding the extra parameters in the H_1_ model, we also changed two population variances of the variables. As a result, the standardized values of the parameters may also change, compromising the interpretation of specified parameter values according to a standardized metric. If one clicks the button that says “View H1 values” in the app, a pop-up window appears that contains the model-implied covariance matrix of the H_1_ model. The variances of the variables are on the diagonal of the covariance matrix. In a path model where all variances equal 1, all parameters are in the standardized metric. In a factor model, the same is true when the common factors are scaled by fixing the factor variances to 1. If the model-implied variances are not equal to 1, users may want to change some population values (for example by increasing or decreasing residual variances) such that the model-implied variances are 1. Users can inspect the table containing the values of the H_1_ parameters in the standardized metric in the pop-up window. In our example, the model-implied variances of EE and DP are no longer exactly 1, but are close enough to ensure that the difference between the standardized values of the added direct effects and the specified values are within rounding error.

**Fig. 3:**
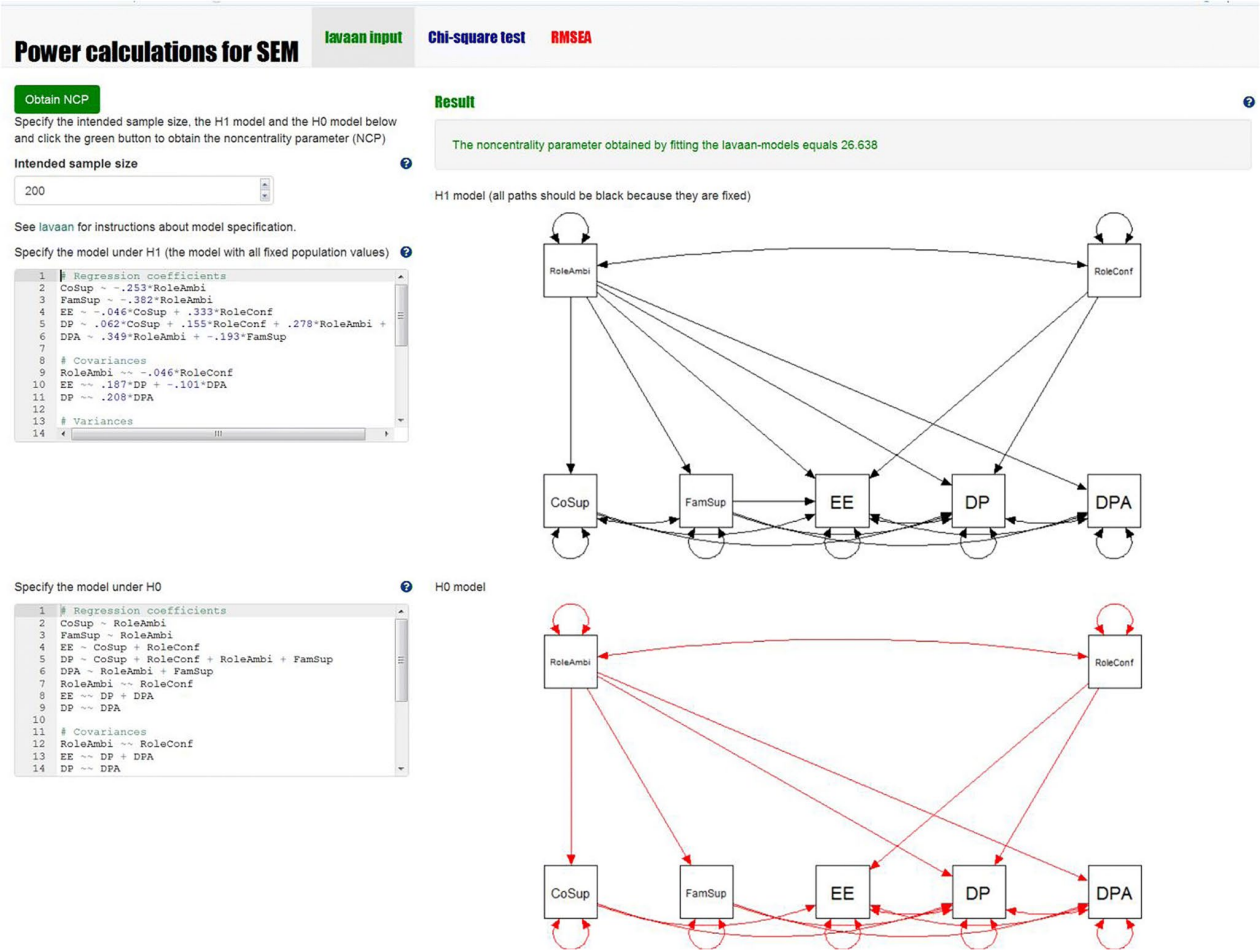
Screenshot of the calculation of the noncentrality parameter in power4SEM

**Fig. 4:**
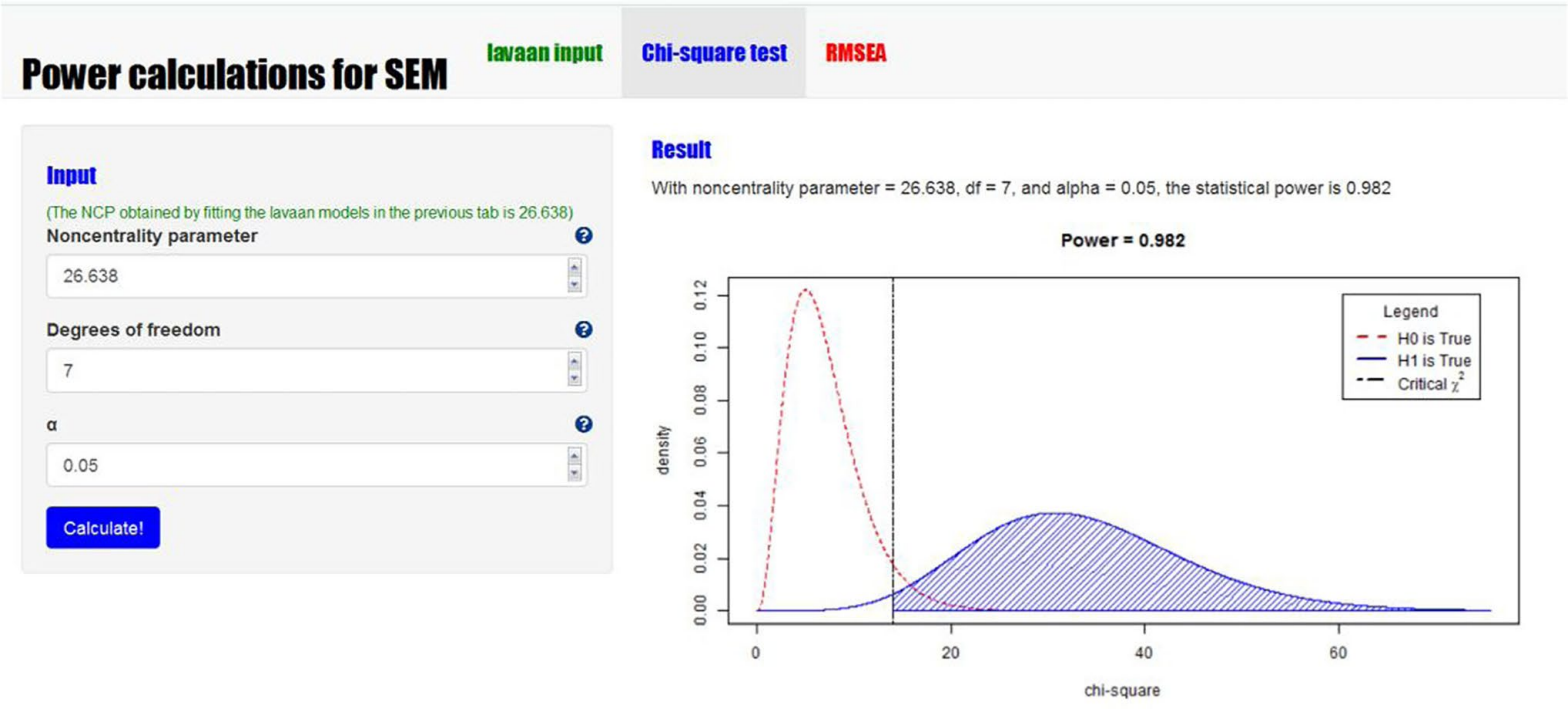
Screenshot of the calculation of the statistical power of the χ2 test in power4SEM

- **Step 2 -** The next step is to specify the model under H_0_. In our app, the lower input box on the left can be used to add the lavaan syntax specifying the model to be tested. A graphical display of the model to be analyzed is shown next to the input box. Since this model contains free parameters, this figure contains red parameters. Figures 3 and 4 show a screenshot of the app with the input boxes and the graphical displays of our example model. If we hit the green button that says “Calculate NCP,” power4SEM will fit the H_0_ model to the population data generated under H_1_, with the specified intended sample size, using the function SSpower() from the semTools package (Jorgensen et al., 2020). The resulting χ^2^ value is the noncentrality parameter that we need to calculate the power. In our example, the noncentrality parameter equals 26.638.
- **Step 3 -** In the second tab of the app, we can calculate the power of the χ^2^ test using the obtained noncentrality parameter. By filling in the noncentrality parameter (λ = 26.638), *df* = 7, and α = .05, the two associated χ^2^ distributions and the calculated power will appear at the right side. In this example, we see that the power to reject the overall fit of the path model, given the chosen H_1_ model, equals .982. At the lower left part of this tab, the minimum sample size that would be needed to obtain a specific power level can be calculated. In this example, a sample of 109 would be needed to obtain a power of .80.

### Theoretical background: Power of the χ^2^ difference test

The χ^2^ statistic can be used to evaluate the overall fit of a model, but it can also be used to test the difference between two nested models with the χ^2^ difference (Δχ^2^) test. For example, one may use the χ^2^ difference test to test whether removing a certain direct effect in a path model leads to significantly worse model fit. A specific model (Model A) is said to be nested within a less restricted model (Model B) with more parameters (i.e., fewer *df*) than Model A, if Model A can be derived from Model B by introducing restrictions only. For example, path model A is nested within path model B by fixing one of the path coefficients in Model B to zero, or by constraining two path coefficients in path model B to be equal to each other. This is known as parameter nesting: any two models are nested when the free parameters in the more restrictive model are a subset of the free parameters in the less restrictive model.

The H_0_ for the χ^2^ difference test is that the difference between the population discrepancy values for the two models (Model A and Model B) is zero: ΔF_0_ = F_0_A_ − F_0_B_ = 0, or in other words that the two models fit equally well. The H_1_ is that the models do not fit equally well, or specifically, that the more restricted Model A fits worse than Model B, so that F_0_A_ − F_0_B_ > 0, or equivalently, ΔF_0_ > 0.

As the test statistic of each of the nested models follows a χ^2^ distribution, the difference in χ^2^ values between two nested models is also χ^2^ distributed:

3 $${\Delta \upchi}^2={\upchi_{\mathrm{A}}}^2-{\upchi_{\mathrm{B}}}^2,$$

with degrees of freedom for the difference equal to the difference in degrees of freedom for the two models:

4 $$\Delta df={df}_{\mathrm{A}}-{df}_{\mathrm{B}}.$$

When Model A and Model B fit equally well in the population (so H_0_ is true), then the models have the same F_0_, leading to the same noncentrality parameter λ, such that Δλ = λ_A_ − λ_B_ = 0. In this case, the Δχ^2^ between the models asymptotically follows a central χ^2^ distribution. Under H_1_, so when the two models do not fit equally well, the noncentrality parameter of the most restricted model will be larger, such that Δλ = λ_A_ − λ_B_ > 0. In this case, under the assumption that neither Model A nor Model B is badly misspecified, the Δχ^2^ between the models asymptotically follows a noncentral χ^2^ distribution with noncentrality parameter Δλ (Steiger et al. 1985). See Table 3 for an overview of the hypotheses, models, and distributions associated with H_0_ and H_1_ of the χ^2^ difference test.

**Table 3:** Overview of the hypotheses, models, and distributions associated with H_0_ and H_1_ of the χ^2^ difference test between two nested models Model A (most restrictive) and Model B (least restrictive)

	H_0_	H_1_
Hypothesis	Δ F_0_ = 0	Δ F_0_ > 0
Fit of Model A and Model B	Model A = Model B	Model A ≠ Model B
Value of noncentrality parameter	Δλ = 0	Δλ > 0
Distribution of test statistic	Central χ^2^	Noncentral χ^2^
Mean of test statistic	Δ*df*	Δ*df* + Δλ

The difference in model fit thus can be tested by comparing Δχ^2^ to a χ^2^ distribution with Δdf, which is called the χ^2^ difference test. If Δχ^2^ is significant, the H_0_ of equal fit for both models is rejected, so the less restrictive Model B should be retained. If Δχ^2^ is not significant, the fit of the restricted model (Model A) is not significantly worse than the fit of the unrestricted model (Model B), so the H_0_ of equal fit cannot be rejected. In this case, the more restricted model (Model A) may be preferred based on the parsimony principle.

Note that because all overidentified models (so all models with *df* > 0) are nested in the saturated model (the model with *df* = 0), the overall (χ^2^) test is actually a special case of the Δχ^2^ test. That is, when Model B is the saturated model, χ_B_^2^ and *df*_B_ are zero, so that Δχ^2^ and Δ*df* are the same as the overall χ^2^ and *df* for Model A.

Power calculations for the χ^2^ difference test are straightforward once the noncentrality parameter Δλ is obtained. Obtaining Δλ involves generating population data from the less restricted Model B. When the more restricted Model A is fitted to these data, the model will not fit perfectly and will yield a nonzero discrepancy value F_0_A_. Fitting Model B to the population data will lead to a perfect fit, so F_0_B_ = 0 and λ_B_ = 0. Therefore, the noncentrality parameter for the χ^2^ difference test equals the noncentrality parameter from Model A: Δλ = λ_A_ − 0 = λ_A_ (MacCallum, Browne & Cai, 2006). In practice, we do not need to fit Model B to the data to verify that it will fit perfectly. Therefore, power calculations for the χ^2^ difference test involve the same three steps as before, with the H_1_ model (used to generate population data) being the Model B with the parameter(s) to be tested, and the H_0_ model (model to be fitted to the population data) being the more restricted Model A.

### Example 2: Calculating the power of the Δχ^2^ test

**Fig. 5:**
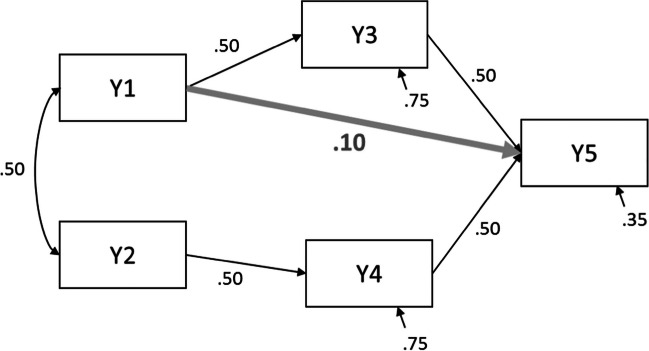
Path model with population values for power calculations in Example 2

Suppose that a researcher wants to know the statistical power of the Δχ^2^ test to detect a direct effect of Y1 on Y5 in the model from Fig. 5. The two nested models that would be compared with a Δχ^2^ test in this case are models with and without estimating the direct effect.

- **Step 1 -** The first step is to calculate the model-implied covariance matrix from the model *with* the direct effect, i.e. the model under H_1_. Similar to the earlier examples, one has to choose population values for each parameter in the model. In this example we chose medium-sized standardized values for the direct effects that are also included in the model under H_0_. We will calculate the power to detect a small standardized effect of .10 of Y1 on Y5. The (residual) variances are chosen in such a way that the total variances of all variables are 1, so that the specified effects are equal to the standardized effects.

- Step 1 consists of calculating the model-implied covariance matrix based on this model. We entered the following code to the first textbox (but see Appendix 2 for the calculation of the model-implied covariance matrix using matrix algebra). Note that paths that are omitted from the specification are path coefficients that are assumed zero in the population, such as the effect of Variable 1 on Variable 4. One can view the model-implied covariance matrix by clicking the button “View H1 values.” The resulting model is graphically shown to the right of the syntax, where all parameters are displayed in black because they are fixed.

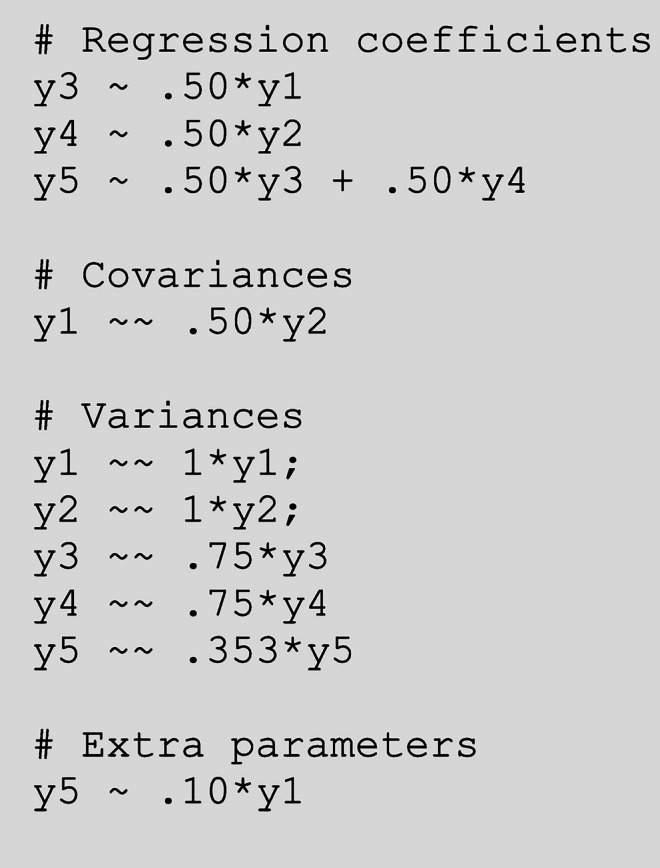

- **Step 2 -** Next, the model under H_0_, which is the model without the direct effect, is fitted to the covariance matrix from Step 1. In the app, the H_0_ model can be specified in the textbox at the lower left side using lavaan syntax^5^. The H_0_ model is the model that does not contain the parameter(s) of interest. So, in our example, the effect of Y1 on Y5 is fixed at zero. Fitting this model to the population data with a certain sample size provides a χ^2^ value, which equals the noncentrality parameter. In this example, the app fits the H_0_ model with *N* = 200, which results in a noncentrality parameter of λ = 4.007. The noncentrality parameter is the misfit that arises because the direct effect of Y1 on Y5 is .10 in the population, but it is not included in model H_0_.
- **Step 3 -** The power of the Δχ^2^ test is calculated by inserting the values of the noncentrality parameter (4.007), the degrees of freedom of the test (1; the difference in the number of parameters between model H_0_ and model H_1_) and the sample size (200) in the second tab of the app. The result then shows that under the specified conditions, the power to detect the effect of Y1 on Y5 equals 52%, which is quite low. With the button at the lower left of this page in the app, one can calculate how large the sample should be to reach different power levels. In this example, one would need a sample size of 391 to obtain 80% power for the Δχ^2^ test.

By calculating the power of the Δ χ^2^ test, we anticipated a situation in which one has an a priori hypothesis about this specific effect, and therefore would test the significance of this specific effect with the Δ χ^2^ test with *df* = 1. Note that the same noncentrality parameter can be used to calculate the power to reject the overall χ^2^ test for exact fit of model H_0_, because the overall χ^2^ test is actually a Δχ^2^ test against the saturated model. In this example the H_0_ model is correctly specified except for one direct effect, because the other parameters that are assumed to be zero in H_0_ are indeed zero in the population. Still, the overall χ^2^ test would have *df* = 5, because it is a test relating to all parameters that are not included in the model, regardless of how many of those parameters are nonzero in the population. In this example, the overall χ^2^ test with *df* = 5 would have 29.2% power to reject exact fit.

## RMSEA-based power

In addition to the χ^2^ statistic, researchers often use the RMSEA to evaluate overall model fit. The RMSEA assumes that the specified model will only be an approximation to reality, and thus some specification error should be allowed. An advantage of using RMSEA-based power calculations is that instead of choosing specific values for all parameters in the H_1_ model, one only needs to choose the RMSEA values related to H_0_ and H_1_. Before introducing power calculations with the RMSEA, we briefly explain how the RMSEA is used in practice.

### Theoretical background: RMSEA-based power

#### The RMSEA and tests of (not-)close fit

The rationale behind the RMSEA measure of fit is that the H_0_ of exact fit (i.e., Σ_population_ = Σ_model_) is invariably false in practical situations. Therefore, the hypothesis of exact fit is replaced by the hypothesis of approximate fit:

$${\Sigma}_{\mathrm{population}}\approx {\Sigma}_{\mathrm{model}},$$

where it is assumed that the specified model will only be an approximation to reality, and thus some specification error should be allowed such that Σ_model_ will never be exactly equal to Σ_population_. The RMSEA is a measure of approximate fit, and is computed based on the sample size, the noncentrality parameter (χ^2^ − *df*), and the *df* of the model. In the formula for the RMSEA, the noncentrality parameter is divided by *df* × *n*, which makes it less sensitive to changes in sample size, and produces a measure of misspecification per *df*. It therefore also takes model parsimony into account. The point estimate of the RMSEA is calculated as follows:

5 $$\mathrm{RMSEA}=\sqrt{\frac{\max \left(\left({\chi}^2- df\right),\kern0.75em 0\right)}{df(n)}}=\sqrt{\frac{\max \left(\hat{\uplambda},\kern0.75em 0\right)}{df(n)}}$$

Note that if χ^2^ < *df*, then the RMSEA is set to zero. An RMSEA of zero indicates that the model fits at least as well as would be expected if the H_0_ of exact fit were true. However, in evaluating the value of the RMSEA, we accept some error of approximation. Browne and Cudeck (1992) suggested that an RMSEA < .05 indicates “close fit,” an RMSEA between .05 and .08 is thought to indicate a “reasonable error of approximation,” and models with an RMSEA above .10 have poor fit. MacCallum, Browne, and Sugawara (1996) suggested that an RMSEA between .08 and .10 indicates mediocre fit.

A confidence interval (CI) can be computed for RMSEA. Ideally, the lower value of the 90% CI includes or is very near zero and the upper value is not very large, i.e., less than .08. Browne and Cudeck (1992) proposed the “test of close fit” where it is tested whether RMSEA is significantly greater than .05 (i.e., the H_0_ is that if we fit our model to the population covariance matrix, RMSEA ≤ .05). We conduct the test by constructing a CI, using a confidence level that is 2 × α (so that we can conduct a one-sided test of our directional hypothesis using the CI). When the lower confidence limit is larger than .05, we can reject the H_0_ of close fit (because the entire CI is above the .05 threshold). MacCallum et al. (1996) extended this idea by “flipping” H_0_ (i.e., that the population RMSEA ≥ .05), which they called a “test of not-close fit.” When the *upper* confidence limit of the RMSEA is *smaller* than .05, we can reject the H_0_ of not-close fit (because the entire CI is below the .05 threshold). The reason that testing not-close fit may be more intuitive is explained by MacCallum et al.:

The test of not-close fit provides for more appropriate roles for the null and alternative hypotheses in the context of model evaluation. When specifying and evaluating a model, our research hypothesis would normally be that the model provides a good approximation to the real-world phenomena under study. As is often pointed out in introductory treatments of hypothesis testing (e.g., Champion 1981), the research hypothesis is most appropriately represented by the alternative hypothesis, so that rejection of the null hypothesis implies support for the research hypothesis. If the research hypothesis corresponds to the null hypothesis, then it becomes very difficult to support the research hypothesis, as is the case in usual tests of model fit in CSM [Covariance Structure Modeling]. (MacCallum et al., 1996, p. 136)

**Fig. 6:**
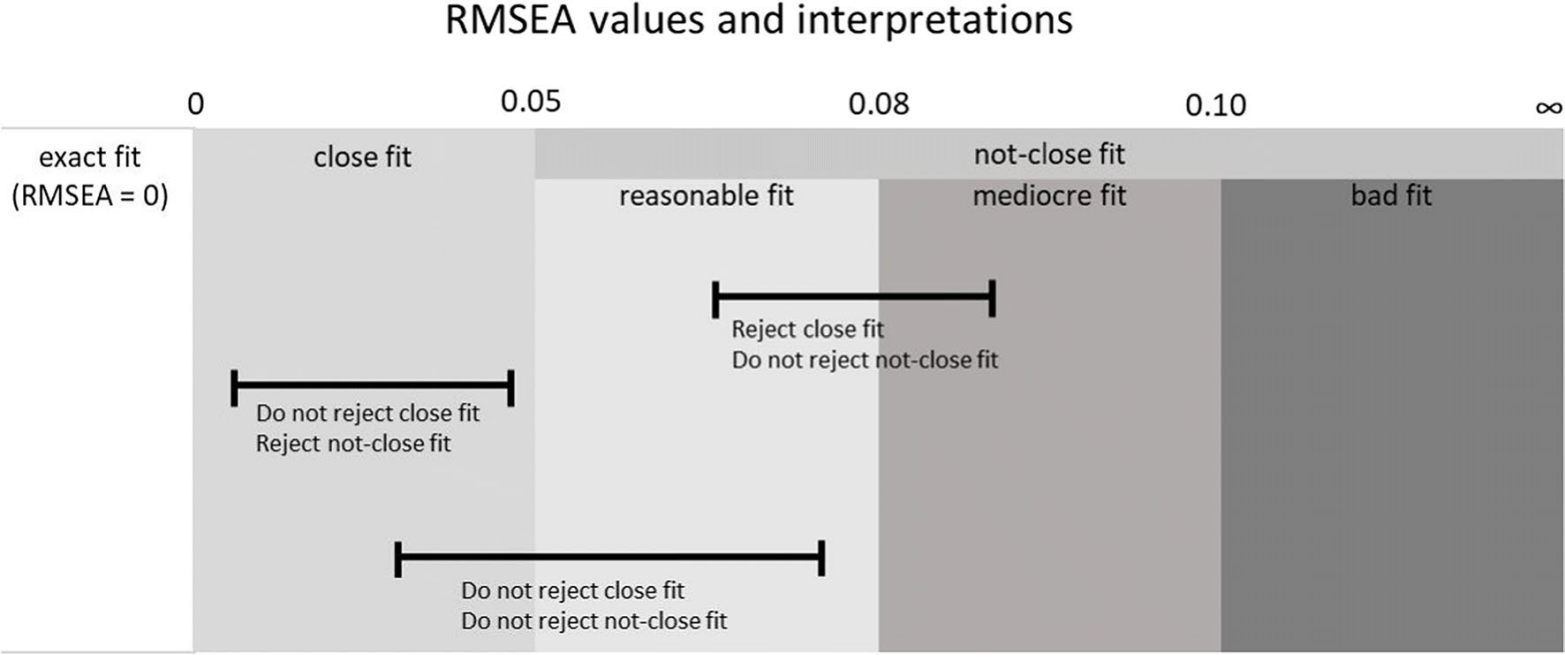
RMSEA values and associated interpretations, with some example confidence intervals and outcome of a test of close or not-close fit

Figure 6 shows an overview of the RMSEA values and associated interpretations, with some example confidence intervals. The first confidence interval lies completely outside the gray area associated with “close fit,” and therefore the hypothesis of close fit will be rejected. The hypothesis of not-close fit will not be rejected, because the confidence interval contains values associated with not-close fit. The second confidence interval falls completely in the area associated with “close fit.” Therefore, the hypothesis of not-close fit would be rejected, and the hypothesis of close fit would not be rejected. The last confidence interval contains values associated with close fit as well as values associated with not-close fit, so neither hypothesis would be rejected.

## Power analysis for the RMSEA test of close fit

MacCallum, Browne, and Sugawara (1996) describe a method to calculate power for SEM, based on the RMSEA. The RMSEA index follows a noncentral χ^2^ distribution. The advantage of power calculations using the RMSEA is that the noncentrality parameter (λ) of the χ^2^ distribution can be derived from the RMSEA by rewriting Eq. 5:

6 $$\uplambda ={\mathrm{RMSEA}}^2\times df(n)$$

**Fig. 7:**
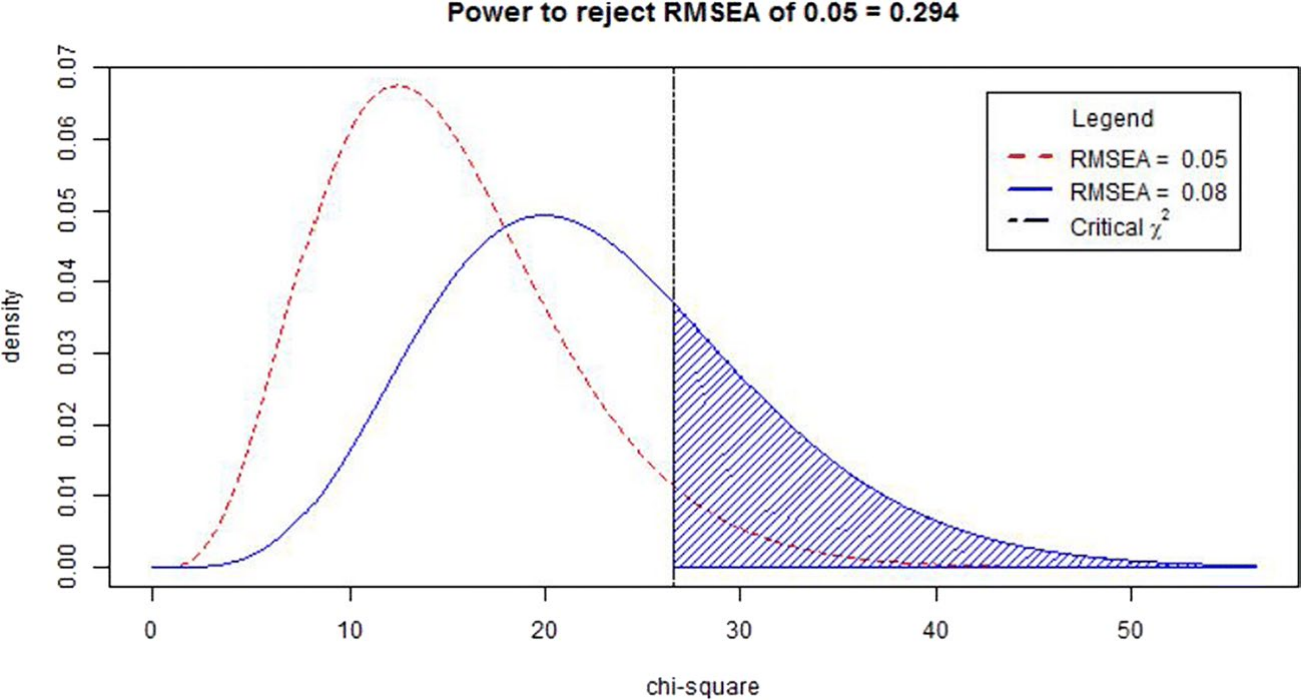
Noncentral χ^2^ distributions related to RMSEAs of 0.05 and 0.08 with *df* = 10 and *N* = 200

Therefore, the noncentral χ^2^ distributions for H_0_ and H_1_ can be easily derived when we use the RMSEA values associated with “close approximate fit” or “reasonable approximate fit,” making power calculations based on the RMSEA relatively simple. MacCallum et al. suggested calculating the power to reject close fit (H_0_: RMSEA ≤ .05) when in the population there is not close fit (H_1_: RMSEA = .08). Figure 7 shows the noncentral χ^2^ distributions related to these two RMSEA values with *df* = 10 and *N* = 200. The vertical dotted line shows the point for which larger observed RMSEA values are associated with χ^2^ values that would lead to rejection of the hypothesis of close fit. The shaded area then shows the area under H_1_, which represents the statistical power.

### Example 3: Power to reject close fit of a longitudinal factor model

**Fig. 8:**
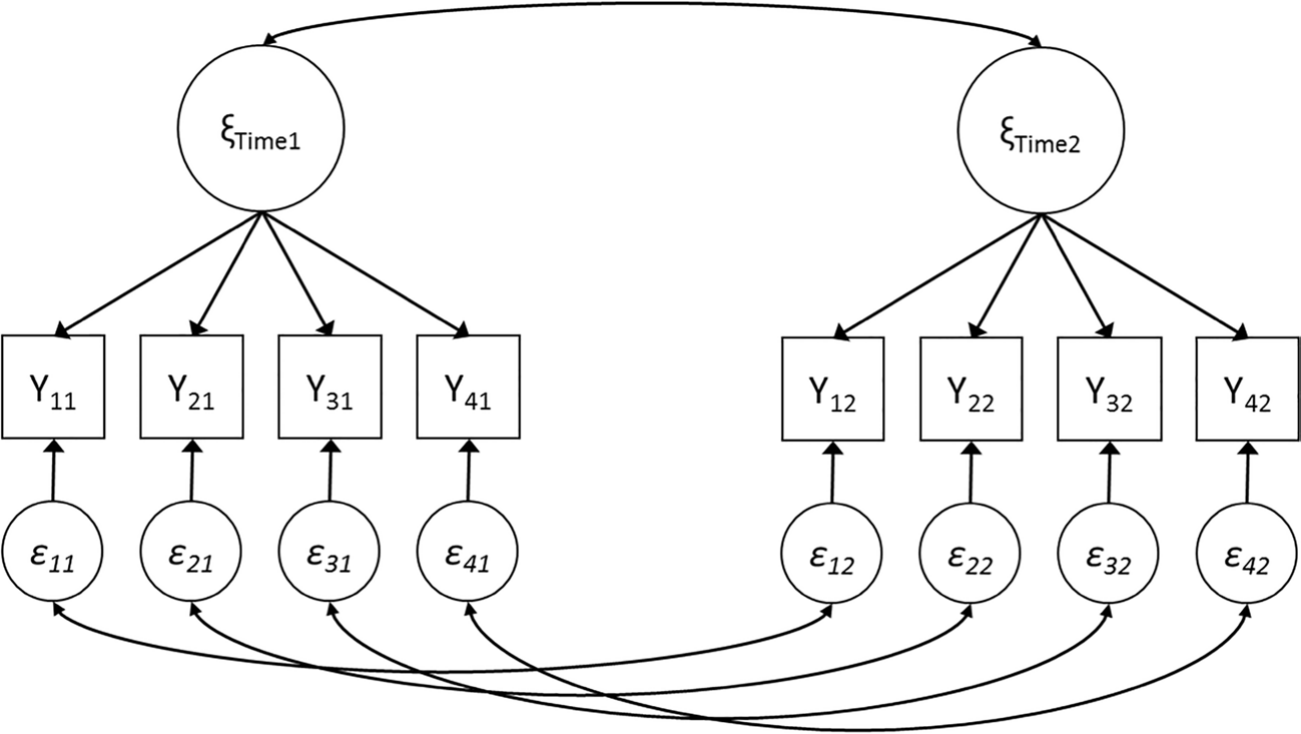
The longitudinal factor model from Example 3

Suppose one wishes to evaluate the power to reject close fit of the longitudinal factor model without means from Fig. 8. This model consists of one factor with four indicators, measured at two time points. With eight observed variables, the number of observed unique variances and covariances is (8 × 9)/2 = 36. In a model without any constrained parameters over time, there will be 21 freely estimated parameters (when scaling by fixing the factor variances: eight residual variances, four residual covariances, eight factor loadings, and one factor covariance). Thus, for this model, *df* = 36 − 21 = 15.

We can use the third tab in the app to calculate the power to reject close fit if in the population there is not-close fit (see the screenshots in Appendix 3). In the left panel we insert the RMSEA value associated with H_0_ (RMSEA = 0.05) and the RMSEA value associated with H_1_ (RMSEA = 0.08). We also fill in the degrees of freedom of the model (15), the intended sample size (*N* = 200), and the α level (0.05). Then, to the right side of the panel we see the two distributions related to H_0_ and H_1_, and the associated power. In this example, the power to reject close fit when in reality there is not-close fit equals 0.378. A power of .378 is generally unacceptable, so based on this result researchers would try to increase the sample to obtain more power. The app indicates that for 0.80 power, one would need a sample size of 551.

## Power analysis for the RMSEA test of not-close fit

**Fig. 9:**
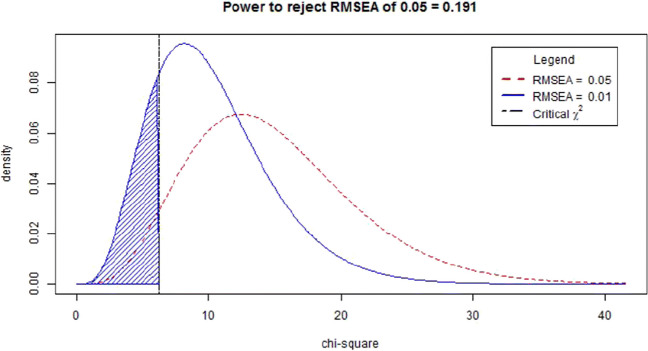
Noncentral χ^2^ distributions related to RMSEAs of 0.05 and 0.01 with *df* = 10 and *N* = 200

In SEM analysis, we hope that the entire confidence interval is below the RMSEA = .05 threshold. It would therefore make more sense to calculate the power to reject a hypothesis of not-close fit in favor of a hypothesis of close fit. When calculating the power of a test of not-close fit, the H_0_ will be that the model does not fit closely (RMSEA ≥ 0.05), and the H_1_ model will be closely fit (for which MacCallum et al. suggest using an RMSEA value of .01). Figure 9 shows the noncentral χ^2^ distributions related to these two RMSEA values with *df* = 10 and *N* = 200. Note that the distribution associated with H_0_ is identical to Fig. 7, but for this test the distribution associated with H_1_, and the area associated with the statistical power, lies on the *left* side of the H_0_ distribution. The interpretation of the power of 0.124 is as follows: if in the population the RMSEA is .01, then the probability of correctly rejecting an H_0_ of RMSEA ≥ .05 equals .124.

### Example 4: Power to reject not-close fit of a full SEM model

**Fig. 10:**
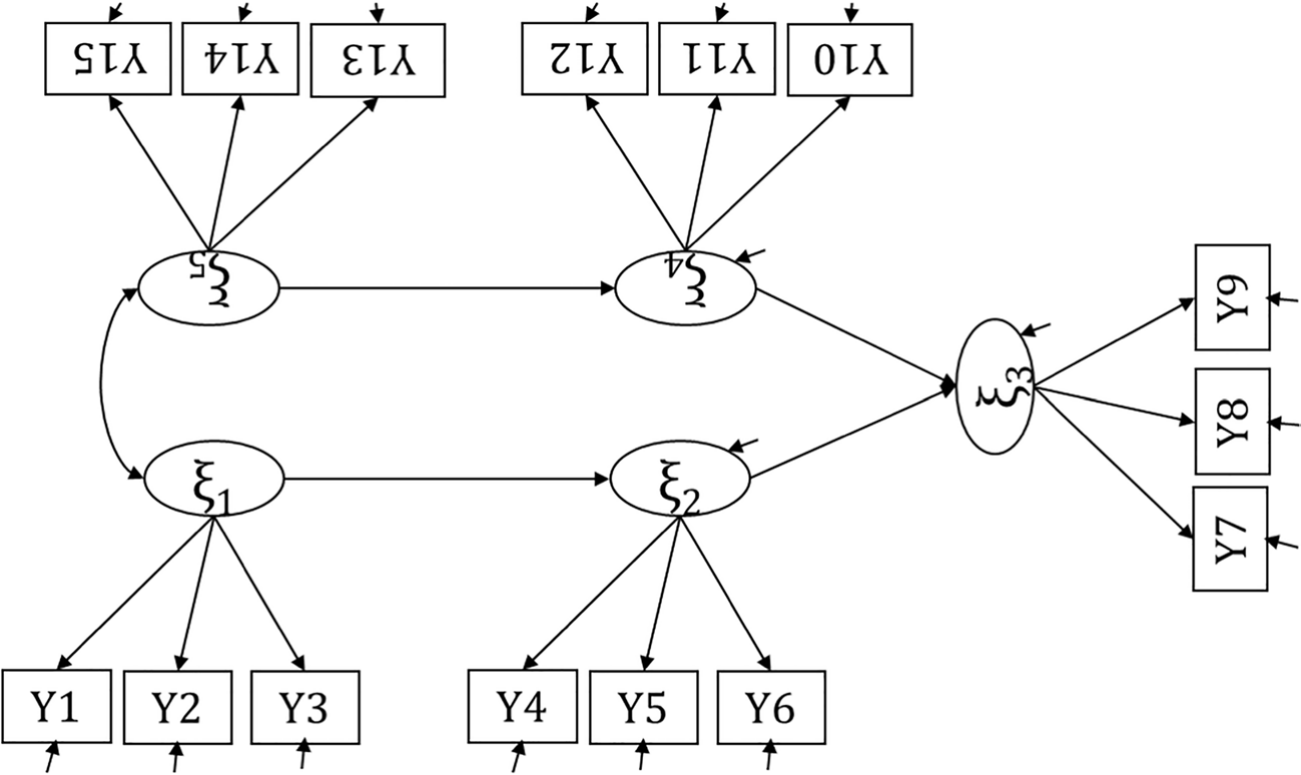
The full SEM model from Example 4

Suppose that one wants to evaluate the power to reject not-close fit of the full SEM model (without means) in Fig. 10. With 15 observed variables, there are 15 × 16/2 = 120 unique observed statistics. The model contains 25 freely estimated parameters, being 15 residual variances of indicators, 10 factor loadings (one factor loading per factor will be fixed for scaling), five (residual) factor variances, one factor covariance, and four direct effects. Therefore, this model has 120 − 35 = 85 *df*.

For the test of not-close fit, we assume a population RMSEA of .01, and we test the H_0_ of RMSEA ≥ .05 with an intended sample size of 200 and an alpha level of .05. The resulting power to reject not-close fit equals 0.816. The app indicates that for a power of 0.80, we would need a sample size of 195.

## χ^2^ -based power with H_1_ based on the RMSEA

As explained before, the χ^2^ test of exact fit assumes that the population value of the RMSEA is zero. This means that one can also calculate the power to reject exact fit using the tab for RMSEA-based power, by setting the RMSEA for H_0_ to zero. The RMSEA value for H_1_ then defines the noncentrality parameter. An advantage of this procedure is that the power of the overall χ^2^ test can be evaluated without specifying population values for all parameters. To illustrate the relation between χ^2^-based power and RMSEA-based power with H_0_ representing zero misspecification in the population, consider the following two examples (where we use α = 0.05 throughout).

The power to reject an H_0_ RMSEA of zero when the H_1_ RMSEA is .08 with *df* = 7 and *N* = 200 equals 0.555. For an RMSEA of .08, the noncentrality parameter λ equals 8.9152, obtained by plugging in 0.08 in Eq. 6. Using this λ in the second tab of the app (again using *N* = 200 and *df* = 7) shows a power of 0.555 to reject the χ^2^ test of overall exact fit. So, for the null hypothesis of exact fit (i.e., RMSEA equals zero), power calculations using the χ^2^ procedure or the RMSEA procedure actually coincide. The difference between the two procedures lies in the way the alternative hypothesis is defined: using an RMSEA value or by defining specific population values for the H_1_ model.

The relation can also be shown the other way around. In Example 1, using a model with *df* = 7 and *N* = 200, the power to reject overall exact fit, obtained by defining the H_1_ model explicitly, was .982. The noncentrality parameter (λ) for this power analysis was 26.638. We can calculate the RMSEA value using this noncentrality parameter using the formula for the RMSEA provided in Eq. 4. The RMSEA value based on this noncentrality parameter is sqrt(26.638/(7 × 199)) = 0.138. Using H_0_ = 0 and H_1_ = 0.138 for the RMSEA-based power calculation again leads to statistical power of .982 to reject exact fit.

## Discussion

In this article we presented a tutorial and app to facilitate power analyses for researchers who plan to use SEM to analyze their data. When designing the app, we aimed at finding a good balance between providing enough functionality to be able to do power analyses, and keeping the app user-friendly and intuitive in use. There are situations in which researchers should use software other than power4SEM for power analyses. These situations are explained below. After that, we discuss some practical issues regarding power analysis for SEM.

## Features that are not implemented in power4SEM

Power4SEM only allows the evaluation of single group models. For power analyses with multi-group models, we advise researchers to use the SSpower() function in the package semTools directly. In this case the function needs a list of population means, a list of population covariances, and vector with sample sizes for each group, and fits the provided H_0_ model to the provided moments for each group. The help page of the function (accessible using the command ?SSpower in R) shows an example of a multi-group SEM power analysis.

The Satorra–Saris method is not suitable for power calculations regarding specific indirect effects. If one wants to obtain the power to detect a nonzero indirect effect in SEM, one should use a Monte Carlo analysis (Zhang, 2014). Schoemann, Boulton, and Short (2017) created a Shiny app to facilitate power analyses for some specific mediation models. Alternatively, one can use WebPower (Zhang & Yuan, 2018) to conduct power analysis for any mediation model.

Our app does not facilitate power analyses for multilevel SEM. We are not aware of software that is specifically designed to do power calculations for multilevel SEM; therefore, to our knowledge, the only option for determining the necessary sample size in such a scenario would be to conduct a Monte Carlo simulation study. The article by Muthén and Muthén (2002) may be useful for setting up such a study.

The R functions behind the app use normal theory maximum likelihood estimation, and therefore assume multivariate normality. If one expects to fit SEMs on non-normal data, one should also conduct a Monte Carlo analysis. WebPower (Zhang & Yuan, 2018) allows one to draw a path diagram for the H_0_ model and the H_1_ model, define the population levels of skewness and kurtosis, and run the Monte Carlo analysis to determine the power or necessary sample size.

The implemented method fits the null-hypothesized model to a covariance matrix to obtain the noncentrality parameter of the χ^2^ distribution pertaining to H_1_. Fitting a model to a covariance matrix assumes a covariance matrix that is calculated from complete data. If researchers expect missing data, they should fit the model on the raw data. Therefore, in order to calculate the power for missing data scenarios, population raw data corresponding to H_1_ are needed. Power calculations for the LRT with data missing completely at random (MCAR) are described by Dolan, van der Sluis, and Grasman (2005). Such population data can be obtained using transformation methods that are described by Bollen and Stine (1993). The difficulty with missing data is that population data need to be generated separately for each group of cases with a different missing data pattern. If there are five variables, there may be 2^5^ = 32 patterns of missingness, each associated with a specific portion of the sample. The sample size of a specific group may be smaller than the number of variables, possibly leading to nonpositive definite covariance matrices in such groups. Moreover, this method is only applicable for data MCAR, which may not be realistic. Therefore, we chose not to implement this method in our app. Researchers who wish to evaluate power for specific missing data patterns may conduct a Monte Carlo simulation instead. Alternatively, a future analytical method might be developed based on similar methods used by Rhemtulla, Savalei, and Little (2016), which (like the Satorra–Saris method) would be less computationally demanding than a Monte Carlo simulation.

## Practical recommendations for power analysis using power4SEM

### Specifying sensible population values

Specifying the values for the population parameters in the H_1_ model for power calculations of χ^2^ tests is probably the hardest part of conducting such a power analysis. A researcher needs to have a feeling for what parameter values are typical for the model and variables under consideration, as well as a clear idea about the number and size of the parameters that should quantify the model misspecification. The general recommendation is that researchers should use all available relevant information to make informed estimates of the parameter values (MacCallum et al., 2006). The available relevant information can for example come from earlier research involving the same (or similar) variables and models, from the analysis of pilot data, or from strong theoretical hypotheses. This implies that χ^2^-based power analysis is most practical for research domains that include a large body of prior research on the topic. In situations where it is impossible to come up with sensible population values for the H_1_ model, one could quantify the misspecification using an RMSEA value, as shown in the last example of this paper.

### Determining which power analysis is needed

Naturally, we recommend conducting a power analysis on the analysis that one will use to answer the research question. To evaluate the exact fit of a hypothesized model, a power analysis concerning the overall χ^2^ test is appropriate. Similarly, to test a hypothesis about the difference between two models, a power analysis for the χ^2^ difference test will be informative. χ^2^-based power results based on explicit choices about parameter values associated with H_1_ are attractive because interpretation of the resulting statistical power is quite intuitive. For example, the power estimate of .70 in Example 1 is directly related to the detection of two direct effects and a covariance that were specified as additional parameters in the H_1_ model. In Example 2, we calculated the power to detect a standardized direct effect of .10 in a specific path model. When H_1_ is not formulated explicitly, but the misfit is based on an RMSEA value, conducting power analyses is easier, but interpretation of the result is less intuitive because the specified misfit is less targeted. For example, obtaining 80% power to detect an overall misspecification as defined by an RMSEA of .08 is less intuitive than obtaining 80% power to detect a specific direct effect of .10.

A drawback of the χ^2^ test of exact fit is that the H_0_ of exact fit will invariably be false in practice, because no model is a perfect representation of reality (Box, 1976). With samples large enough to have large power, models that are only wrong to an irrelevant degree will be rejected by the χ^2^ test. Therefore, many researchers focus on approximate fit indices.

We recommend that if a researcher intends to use the RMSEA to judge model fit, then RMSEA-based power calculation is needed. Given the relative simplicity of the procedure, we recommend power analyses for both the test for close fit and the test for not-close fit. When researchers intend to use different RMSEA values for the evaluation of model fit from those used in this tutorial, then the RMSEA values associated with H_0_ and H_1_ can be changed accordingly. For example, when a researcher is satisfied with the model when the RMSEA value is below .08 instead of .05, they could do a power analysis where the RMSEA for H_0_ represents bad fit (say, RMSEA = .12), and RMSEA for H_1_ equals .08. This leads to a power estimate of the rejection of bad fit when in reality there is mediocre fit.

## Conclusion

Conducting a power analysis for SEM is not easy. With this tutorial and with the Shiny app power4SEM, we try to facilitate the statistical part of conducting a power analysis. However, probably the most difficult aspect of doing a power analysis is that it requires careful thinking about the hypotheses to test, the parameter values one expects, and the questions that need to be answered. Although this may seem to be a drawback of power analysis, it is of course a good thing in itself if researchers think about their analysis plan carefully before collecting data. Moreover, a carefully conducted power analysis will prevent wasting expensive resources on under- or overpowered studies.

## Appendix 1

Calculating the model-implied population covariance matrix under H_1_ of Example 1

## Appendix 2

Calculating the model-implied population covariance matrix under H_1_ of Example 2

## Appendix 3

Screenshots of power4SEM for all examples and one additional example

### Example 2: Calculating the power of the Δχ^2^ test

Obtaining the noncentrality parameter:

Calculating power and minimum sample size:

### Extra example: Calculating the power of the χ^2^ test for overall fit for a latent growth model

Suppose one is interested in calculating the power of the overall χ^2^ test for a linear growth curve model on four measurements. This model has five degrees of freedom, so with an α-level of 0.05, exact fit of the H_0_ model would be rejected if the observed χ^2^ were larger than 11.071. The H_1_ model is defined as a specific just-identified model, where one again has to choose values for *all* population parameters (but one can still assume population values of zero for parameters). In this example we specify small nonzero residual covariances between adjacent time points and zero covariance between nonadjacent time points. In addition, we specify nonzero intercepts at year 3 and year 4, leading to nonlinear growth instead of linear growth. The lavaan syntax for the H1 model is then:

**Step 1 –** The figure below shows the part of the Shiny app where we entered the lavaan syntax for the H_1_ model in the upper left textbox. The app then provides a graphical display of the model next to the syntax. In the graphical display, all fixed parameters are represented in black, and all free parameters are in red. In the population model under H_1_, all parameters should be specified as fixed values, so all parameters in the graph should be black.

**Step 2 -** The textbox at the lower left part contains the syntax for the H_0_ model, which contains free parameters. In this case, the estimated parameters are the growth factor means, variances, and covariance, and the residual variances of the indicators. The graphical display at the right side shows the freely estimated parameters in red. Note that the residual covariances that were specified to be present in the population (under H_1_) are not estimated in the model under H_0_.

In this example the noncentrality parameter based on *N* = 200 is 11.52. Given this noncentrality parameter, we know that under H_1_, the test statistic follows a noncentral χ^2^ distribution, with *df* = 5 and λ = 11.52.

**Step 3 -** The power is found by determining the area under the H_1_ distribution that lies to the right of the critical value under the H_0_ distribution. For a central χ^2^ distribution with *df* = 5 and α = .05, the critical value is 11.07. In the second tab of the app, one can provide all necessary information on the left side, and then one will see the resulting power and the associated χ^2^ distributions on the right side.

In this example, the power to reject exact fit is .749. The app also lets researchers calculate the minimum sample size needed to obtain a desired power level. In this example, we would need *N* = 223 obtain 0.80 power.

## Footnotes

1 The R code needed to run the app locally is available from https://osf.io/39gx8/

2 For more information on how to specify models in lavaan see http://lavaan.ugent.be/tutorial/.

3 We used the conventional values proposed by Cohen (1988, 1992) to represent small, medium, and large effect sizes throughout this tutorial. However, as Cohen also cautioned, values that should qualify as small, medium, or large effects depend on the research domain. For example, appropriate values are found to be smaller than Cohen’s values in organizational psychology (Bosco et al. 2015) and social psychology (Lovakov & Agadullina, 2017).

4 In analyses without mean structure, it is also possible to use Wishart likelihood, in which case *n* = *N* − *G*, where *G* is the number of groups. Wishart likelihood is the default in older SEM software (LISREL and EQS), but not in lavaan, which our Shiny app uses.

5 To limit the number of figures, we do not provide screenshots of the app for all examples in the article itself, but screenshots for Examples 2–4 can be found in Appendix C. The appendix also contains an additional example of a power calculation for a latent growth model.
