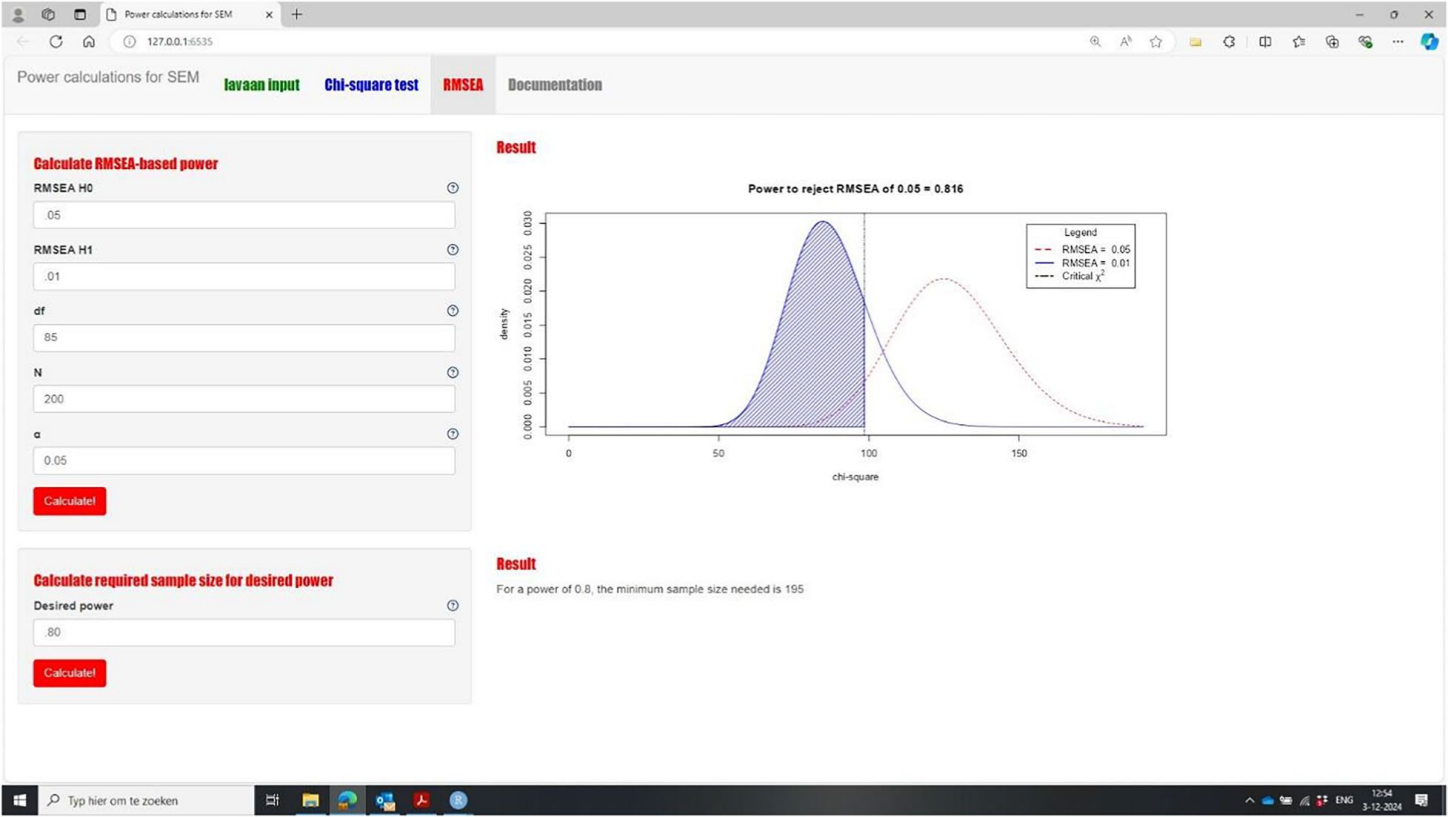# Author Correction: Analytical power calculations for structural equation modeling: A tutorial and Shiny app

**DOI:** 10.3758/s13428-024-02571-5

**Published:** 2025-01-09

**Authors:** Suzanne Jak, Terrence D. Jorgensen, Mathilde G. E. Verdam, Frans J. Oort, Louise Elffers

**Affiliations:** 1 Methods and Statistics, Research Institute of Child Development and Education, University of Amsterdam, Nieuwe Achtergracht 127, 1018 WS Amsterdam, The Netherlands; 2 Methodology and Statistics, Institute of Psychology, Leiden University, Leiden, The Netherlands; 3 Educational Sciences, Child Development and Education, University of Amsterdam, Amsterdam, The Netherlands

**Author Correction: Behavior Research Methods (2020) 53:1385-1406**

10.3758/s13428-020-01479-0

The original online version of this article was revised:

The following sentences from page 1396 were updated as indicated. The model contains 25 freely estimated parameters. Was updated to: 35 freely estimated parameters.

Therefore, this model has 120 − 25 = 95 *df*. Was updated to: 120 - 35 = 85 *df*.

The resulting power to reject not-close fit equals 0.854. Was updated to: 0.816

The app indicates that for a power of 0.80, we would need a sample size of 183. Was updated to: 195.

The figure in **Example 4** has been corrected to show the changes made by the updates.